# Interruptions of enteral nutrition in intensive care units: mechanisms, clinical impacts, and precision nursing interventions–a scoping review

**DOI:** 10.3389/fnut.2026.1768983

**Published:** 2026-05-28

**Authors:** Quanquan He, Jiani Qian, Maoyun Miao, Xiaowei Liu, Weiwei Ni, Yulei Ma

**Affiliations:** 1 School of Nursing, Shandong Second Medical University, Weifang, China; 2 Weifang Key Laboratory for Clinical Nursing Research, Department of Spinal Surgery, Affiliated Hospital of Shandong Second Medical University, Weifang, China; 3 Weifang Key Laboratory for Clinical Nursing Research, Department of Nursing, Affiliated Hospital of Shandong Second Medical University, Weifang, China

**Keywords:** enteral nutrition, intensive care unit, nursing interventions, nutrition interruption, precision nursing

## Abstract

**Background:**

Enteral nutrition (EN) is the preferred nutritional support for critically ill patients in intensive care units (ICU). Despite its benefits, enteral nutrition interruption (ENI) occurs frequently in ICU practice and may contribute to inadequate nutrient delivery, which has been associated with poorer nutritional status and potentially unfavorable clinical outcomes. A consolidated synthesis of its mechanisms, clinical impact, and precision nursing interventions remains limited.

**Objective:**

This scoping review aimed to summarize the mechanisms underlying ENI, evaluate its impact on ICU patient outcomes, and identify nurse led precision interventions to optimize nutritional support.

**Methods:**

This scoping review followed JBI methodology, the Arksey and O’Malley framework, and PRISMA ScR standards. Using the PCC structure of ICU patients, ENI, and nursing practice, a three stage search was conducted in major databases up to April 2025. Eligible evidence included experimental, observational, as well as guidelines and implementation projects. Study selection and data extraction were independently completed by two reviewers, with findings synthesized narratively.

**Results:**

29 studies from diverse ICU settings were included. Procedural and diagnostic activities accounted for 63.4 percent of ENI, followed by gastrointestinal intolerance at 19.2 percent, tube related complications at 9.3 percent, and hemodynamic instability at 3.9 percent, while patient and provider related factors contributed approximately 33 percent. ENI was associated with nutritional inadequacy in 38–78 percent of patients and with impaired intestinal barrier function and increased infection risk. Experiencing three or more interruptions has been associated with a higher mortality risk. Precision nursing interventions, including standardized feeding protocols, continuous infusion, reduced gastric residual volume monitoring, perioperative nutritional optimization, and prokinetic agent use, reduced interruption frequency and duration and improved energy and protein target achievement from 26.9 and 18.6 percent to 57.0 and 57.4 percent.

**Conclusion:**

Enteral nutrition interruption is common in ICU practice and has been associated with inadequate nutritional delivery and potentially adverse outcomes in critically ill patients. Nurse-led precision interventions may help reduce interruptions and improve nutritional adequacy, although their effects on broader patient-centered outcomes require further study. Future efforts should prioritize standardized protocols, multidisciplinary collaboration, and intelligent monitoring to advance evidence informed nutritional nursing practice.

## 1 Introduction

Enteral nutrition is a cornerstone of nutritional support for critically ill patients in ICU. By providing essential energy and nutrients, EN may help support immune function, modulate inflammatory responses, and reduce skeletal muscle catabolism, while contributing to tissue repair and the maintenance of gastrointestinal and respiratory mucosal barrier integrity (1–3). There is evidence suggesting that adequate EN may be associated with a reduced incidence of infection complications, shorter hospital stay in ICU, lower potential mortality rates, and improved long-term functional recovery (2, 4, 5). Compared with parenteral nutrition, EN is preferred for ICU patients due to its superior safety profile, lower cost, and beneficial effects on gastrointestinal function (1). However, in clinical practice, ICU patients often fail to reach the preset nutritional targets. Surveys have shown that the actual nutritional intake is only about 60% of the target amount (6). ENI is the main obstacle causing insufficient calorie and protein intake (7). The present review focuses specifically on ENI rather than the general benefits of EN itself.

There is currently no universally accepted operational definition of ENI. Across the included studies, ENI was defined using heterogeneous criteria. Some studies defined ENI based on interruption frequency, considering any single interruption in ENI delivery as an interruption event (8). Other studies defined ENI based on duration, although the minimum threshold varied. A commonly reported criterion is an interruption lasting ≥1 h during continuous EN infusion. For intermittent feeding regimens, ENI may be defined as failure to deliver the scheduled feeding volume during the planned feeding period (9). In this scoping review, trophic feeding was not treated as a separate ENI category unless the original study explicitly distinguished trophic feeding from standard EN delivery. Reported ENI incidence varies widely across studies (4.7%–100%), largely due to heterogeneity in operational definitions, ICU populations, and feeding regimens. For example, one systematic review reported a mean ENI incidence of 48.3% and approximately 2–3 interruptions per patient with a cumulative interruption time of about 33.8 h; these values should be interpreted as descriptive summaries from that synthesis rather than pooled estimates generated by the present scoping review (10). The main reasons for interruption include diagnostic procedures, gastrointestinal intolerance, feeding tube problems, hemodynamic instability, and patient and nursing-related factors. Among these, interruptions due to imaging examinations or surgical procedures accounted for approximately 63.4%, those caused by gastrointestinal intolerance such as gastric retention or diarrhea accounted for 19.2%, problems related to feeding tubes such as blockage or detachment accounted for 9.3%, interruptions caused by hemodynamic instability accounted for 3.9%, and interruptions related to patients or other nursing factors accounted for approximately 33% (10, 11). Frequent interruptions may be associated with inadequate nutritional delivery and an increased risk of malnutrition, and may potentially contribute to unfavorable clinical outcomes in critically ill patients (12, 13). A prospective study reported an association between ≥3 cumulative interruptions and a higher risk of mortality (adjusted odds ratio 6.73), as well as a shorter survival time (14). Furthermore, interruptions have been suggested to be associated with intestinal barrier impairment, alterations in gut microecology, and systemic metabolic disturbances, which may be linked to the development of multiple organ dysfunction syndrome (15).

Although the negative impacts of ENI have received widespread attention, the existing research has significant limitations. Firstly, the definition and evaluation criteria of ENI have not been unified. Different studies adopt different determination standards, such as the frequency of interruptions, duration, or differences in GRV, which lead to limited data comparability (10). Secondly, the existing literature mainly focuses on the causes of interruptions and their clinical consequences, lacking a systematic summary of precise nursing intervention measures, especially in the specific guidance for ICU nursing practices (16–18). As the primary role in the execution and monitoring of EN, nurses are responsible for daily intake verification, identifying risks of interruption, coordinating a multidisciplinary team including nutritionists and doctors to communicate feeding plans, and implementing intervention measures such as adjusting feeding methods or applying stimulant drugs (19). However, the evidence regarding the role of nurses in ENI management and the effectiveness of their interventions is rather scattered, failing to fully reveal how to reduce interruptions by optimizing the nursing process, enhancing professional knowledge, and strengthening team collaboration (20, 21). Furthermore, current research pays less attention to the interruption characteristics of different ICU subgroups, such as surgical, neurological, and burn patients, as well as the targeted nature of nursing interventions. This has limited the development and promotion of standardized protocols. These knowledge gaps restrict the standardization of ENI management and urgently require the integration of evidence-based data to guide ICU nursing practices.

Precision nursing refers to targeted nursing interventions based on individual patient characteristics, evidence-based data, and dynamic monitoring. Its aim is to optimize the continuity of EN through standardized feeding pathways, dynamic nutritional monitoring, and multidisciplinary collaboration, thereby reducing the frequency and duration of interruptions (22). This study adheres to the JBI guidelines and the Arksey and O’Malley framework, adopts the PRISMA-ScR reporting standard, and uses the scope review method to systematically explore the occurrence mechanism, clinical impact, and precise nursing intervention measures of ENI in critically ill patients in the ICU. This scoping review aims to: (1) clarify the complex mechanism of ENI, including the main causes such as diagnostic and therapeutic operations, gastrointestinal intolerance, and their interactions; (2) assess the multi-dimensional impact of interruptions on patients’ prognosis, such as malnutrition, infection risk, and mortality rate; (3) explore the precise nursing intervention strategies led by nurses, including standardized feeding pathways, continuous infusion, etc., to reduce interruptions and improve the rate of nutritional attainment. The research results will provide evidence-based practice guidance for ICU nurses, optimize the nutritional support process, promote multidisciplinary collaboration, and provide directions for developing standardized intervention protocols and intelligent monitoring technologies. This scoping review highlights the core role of nurses in EN management, fills the knowledge gap in the nursing perspective in existing literature, and provides theoretical and practical support for improving the survival quality and clinical outcomes of critically ill patients.

## 2 Methods

### 2.1 Protocol and study design

This scoping review was conducted to map the available evidence on ENI among critically ill patients in ICU. The review focused on the causes and mechanisms of ENI, its associated clinical outcomes, and nurse-led precision nursing interventions. The methodology followed the recommendations of the Joanna Briggs Institute for scoping reviews and was informed by the five-stage framework proposed by Arksey and O’Malley. Reporting was guided by the Preferred Reporting Items for Systematic Reviews and Meta-Analyses extension for Scoping Reviews (PRISMA-ScR).

A scoping review approach was selected due to the complex and multifactorial nature of ENI and the heterogeneity of existing evidence across clinical populations and study designs. Eligibility criteria were defined to include studies reporting on the occurrence, mechanisms, clinical consequences, or nursing-related management of ENI. For the purpose of this review, there is currently no universally accepted operational definition of ENI. Definitions varied across included studies. Some studies defined ENI based on interruption frequency and considered any interruption in EN delivery as an event. Others used duration-based criteria, most commonly an interruption lasting at least 1 h during continuous EN infusion. For intermittent feeding, ENI was defined as failure to deliver the scheduled feeding volume within the planned feeding period. These variations contribute to heterogeneity in reported ENI incidence across studies.

### 2.2 Research questions

This scoping review was guided by the Population, Concept, and Context framework. The population of interest comprised critically ill adult patients admitted to ICU, including clinical subgroups such as surgical, neurological, burn, and trauma patients with varying levels of disease severity and nutritional risk. The concept of interest was ENI, encompassing its mechanisms of occurrence, clinical impact, and management strategies, including standardized feeding pathways, continuous feeding approaches, and precision nursing interventions. The context focused on nursing practice in the intensive care setting, emphasizing the role of nurses in the delivery and monitoring of EN and in multidisciplinary collaboration with physicians and dietitians.

Based on this framework, the review addressed the following research questions:

1. What causes and mechanisms of ENI have been reported among critically ill patients in ICU?
2. What clinical outcomes are associated with ENI, including malnutrition, infection risk, and mortality?
3. What nurse-led precision nursing interventions have been reported to reduce ENI and improve nutritional adequacy?

### 2.3 Selecting the literature (i.e., inclusion-exclusion criteria)

The articles included in this scoping review met the specified inclusion criteria and were carefully chosen to ensure the relevance of the included studies.

Studies were included if they met the following criteria: (1) ≥18 years ICU adult patients, including surgical, neurological, trauma, burn, and respiratory subgroups; (2) Studies addressing ENI mechanisms, clinical impacts, or nurse-led interventions; (3) Study design: randomized controlled trials (RCTs), quasi-experimental studies, observational studies, clinical guidelines, and evidence-based practice or quality improvement projects, all of which were full-text publications from peer-reviewed journals; (4) Language: English; (5) Publication time: Up to April 2025, with no lower time limit.

Studies were excluded if they: (1) Studies conducted outside the ICU; (2) Animal experiments, case reports, conference abstracts, commentaries, review; (3) Studies unrelated to ENI.

### 2.4 Search strategy

The search for primary studies and other eligible evidence sources (e.g., clinical guidelines and quality-improvement/implementation projects) used a comprehensive three-step strategy. Review articles were scanned only to identify additional primary studies via reference-list checking and were not included as evidence sources in the scoping-review synthesis. Initial search: A preliminary search was conducted in PubMed and CINAHL using keywords such as *“enteral nutrition interruption”* and *“interruption”* combined with MeSH terms (*“Enteral Nutrition”*). The retrieved studies were analyzed based on their titles, abstracts, and index terms, which informed the development of a customized search strategy for each database. Comprehensive search: a full search was conducted across PubMed, Web of Science, Cochrane Library, JBI, CINAHL, Embase, and Scopus, covering literature up to April 2025. We focused on papers written in English. A sample PubMed search string was:

(“enteral feed” OR “enteral nutrition” OR “gastric tube feeding” OR “gastrostomy nutrition” OR “intestinal feeding” OR “jejunal feeding” OR “nasogastric feeding” OR “tube feeding”) AND (“cessation” OR “discontinue*” OR “interrupt*” OR “feeding break*” OR “withhold*”). The full search strategies are provided in the Supplementary material.

Reference list search: the reference lists of all the included studies were screened for supplementary evidence.

All search results were imported into EndNote for deduplication. Two reviewers independently screened titles, abstracts, and full texts, with discrepancies resolved by consensus or consultation with a third reviewer.

### 2.5 Data extraction and analysis

**FIGURE 1:**
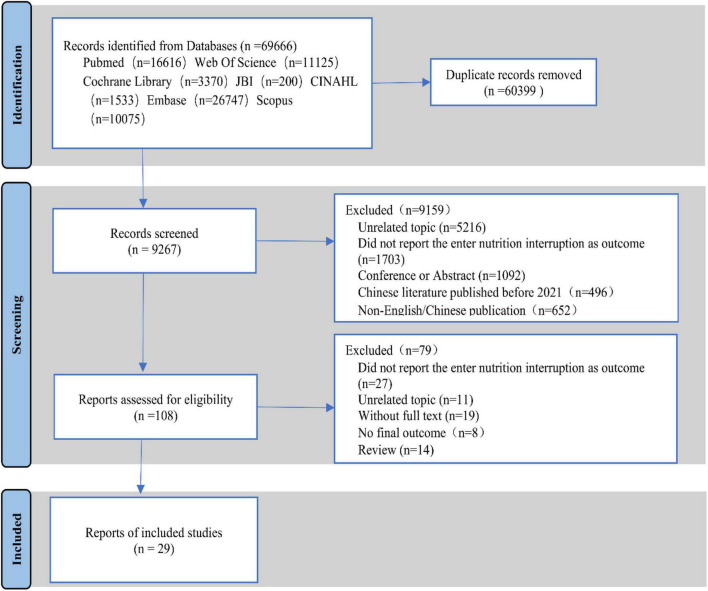
PRISMA flow chart showing study selection for the scoping review.

Two reviewers independently extracted data using a customized Excel form. Extracted information included study characteristics such as author, year, country, study design, sample size, and ICU type, as well as ENI characteristics including ENI definition, threshold, causes, incidence, frequency, and duration. Reported clinical outcomes included malnutrition, infection, mortality, length of stay, and intestinal barrier integrity. Data on nursing related interventions were also recorded, including intervention type, the role of nurses in implementation, and reported nutritional outcomes such as caloric and protein adequacy rates. Special attention was given to the role of nurses in intervention delivery, including aspects such as nurse education, feeding logs, and multidisciplinary coordination. The data were synthesized narratively and categorized under mechanisms, impacts, and interventions, supported by Tables 1A–C (Characteristics of included studies on enteral nutrition interruption in intensive care units) and a PRISMA-ScR flow diagram (Figure 1).

**TABLE 1:** Characteristics of included studies on enteral nutrition interruption in intensive care units (*n* = 29).

References	Country	Study design	Sample	Intervention(s)	Key outcomes	ENI definition	Threshold	Planned vs. unplanned	Avoidable vs. unavoidable	Procedural vs. intolerance-related
A. Clinical guidelines (n = 4).										
Singer et al. (2)	Multi-country	Guideline	ICU patients	ICU stay > 48 h → nutritional risk; start EN.	Potential benefits of early EN (<48 h) on outcomes	NR	NR	Unplanned: hemodynamic instability, high GRV	Unavoidable: hemodynamic instability, high GRV	Intolerance: high GRV
Singer et al. (1)	Multi-country	Guideline	ICU patients	Early EN, risk assessment	Early EN beneficial; adjust per condition	NR	NR	Unplanned: shock, GI bleeding, high GRV, obstruction, ACS	Unavoidable: shock, GI bleeding, obstruction, high GRV, ACS	Intolerance: GI bleeding, high GRV, obstruction
Reintam Blaser et al. (23)	Multi-country	Guideline	ICU requires	Early EN recommended; delay in shock	Recommend early EN, delay EN in specific situations	NR	NR	Unplanned: shock, hemodynamic instability, GI bleeding, high GRV	Unavoidable: shock, hemodynamic instability, High GRV	Intolerance: high GRV, GI bleeding
McClave et al. (3)	USA	Guideline	Adult ICU patients	Early EN and optimized nutrition protocols	Early EN improves outcomes; protocol optimization is needed to reduce ENI	NR	NR	Unplanned: hemodynamic instability, GI/metabolic issues	Unavoidable: hemodynamic instability	Intolerance: GI/metabolic issues
B. Interventional/projects (n = 5).										
Barhorst et al. (24)	USA	Evidence-based practice project	Neurological ICU patients	Implement guidelines, use reminder cards, and nasogastric tube supply kits.	EN ratio rose from 39% to 78% in 48 h; nasogastric tube placed within 21 h on average.	NR	NR	Unplanned: feeding intolerance, disease severity Planned: nursing practices	Avoidable: nursing practices Unavoidable: disease severity	Procedural: nursing practices Intolerance: EN intolerance
Holyk et al. (25)	USA	Retrospective quasi-experimental	*n* = 189 ICU patients	Volume-based feeding (VBF) vs. rate-based (RBF)	VBF ↑ calorie (75% →102%) and protein (68% → 87%)	NR	NR	Planned: dietary restrictions Unplanned: aspiration risk	Avoidable: dietary restrictions, aspiration risk	Procedural: dietary restrictions Intolerance: aspiration risk
Williams et al. (26)	Australia	Before–after	*n* = 653 ICU (excl. cardiac surgery)	Nurse-led multidisciplinary intervention	ENI episodes ↓ from 907 to 662; GI-related ENI ↓ from 14% to 10%	Interruptions to continuous EN	NR	Unplanned: GI problems, tube issues	Avoidable: tube issues Unplanned: GI problems	Procedural: tube issues Intolerance: GI problems
B. Interventional/projects (n = 5).										
Zheng et al. (27)	China	Secondary analysis (multicenter RCT)	*n* = 1331 ICU patients	Stability check before feeding, GI-based feeding plan, GRV algorithm revision, nurse training	Incidence 18.6%; procedures main cause; ENI ↓ energy/protein intake	ENI >30 min.	>30 min	Planned: procedures Unplanned: EN intolerance, hemodynamic instability	Avoidable: tube issues Unavoidable: hemodynamic instability, EN intolerance,	Procedural: procedures, tube issues Intolerance: EN intolerance
Zhu et al. (28)	China	RCT	*n* = 78 cerebral hemorrhage	Continuous vs. intermittent feeding	Continuous ↓ diarrhea, intolerance; calorie intake NS	NR	NR	Unplanned: diarrhea, EN intolerance	Unavoidable: diarrhea, EN intolerance	Intolerance: diarrhea, EN intolerance
C. Observational studies (n = 20).										
Kasti et al. (18)	Greece	Prospective study	*n* = 81 ICU patients	Stop GRV monitoring, multidisciplinary plan	Median ENI 5.2 h/day; energy 1037 kcal/day (target 1751), protein 0.43 g/kg/day (target 1.3)	All interruption events recorded	NR	Planned: procedures, GRV monitoring	Avoidable: GRV monitoring Unavoidable: procedures	Procedural: procedures, GRV monitoring
van Nieuwkoop et al. (29)	Netherlands	Prospective observational	*n* = 61 ICU patients	Shortened-fasting protocol	ENI most frequent first 3 days; 25% not meeting goals in first 4 days	NR	NR	Planned: diagnostic/ endoscopic procedures	Unavoidable: diagnostic/ endoscopic procedures	Procedural: diagnostic/ endoscopic procedures
Onuk et al. (14)	Turkey	Prospective observational	*n* = 122 critically ill	Standard EN protocol	2.74 ENI/patient; total 960 min; ≥3 ENI ↑ mortality	NR	NR	Planned: radiology, surgery Unplanned: tube failure	Avoidable: tube failure	Procedural: radiology, surgery, tube failure
Dorken Gallastegi et al. (30)	USA	Retrospective	*n* = 1701 ICU patients	Early vs. late EN	Early EN ↓ LOS, RRT need, electrolyte abnormalities	NR	NR	Unplanned: hemodynamic instability	Unavoidable: hemodynamic instability	NR
Habib et al. (17)	Pakistan	Prospective observational	ICU patients	Early EN, prone position feeding	Tracheostomy prolonged ENI; prone tolerated well	NR	NR	Planned: pre-op fasting, prone position, tracheostomy	Avoidable: pre-op fasting Unavoidable: tracheostomy	Procedural: tracheostomy, pre-op fasting, prone
C. Observational studies (n = 20).										
Salciute-Simene et al. (31)	Lithuania	Prospective observational	*n* = 73 ICU on EN	Shortened fasting protocol	ENI in 68%; target achievement 26% (ENI days) vs. 45%	Interruption or rate reduction of EN for >60 min.	>60 min	Planned: tracheostomy, surgery Unplanned: hemodynamic instability, high GRV	Unavoidable: hemodynamic instability, high GRV	Procedural: tracheostomy, surgery Intolerance: high GRV
Kozeniecki et al. (32)	USA	Retrospective cohort study	78 MICU patients	Standardize EN; anticipate ENI; compensate via rate/volume-based feeding.	EN start/promotion are main ENI causes; processes need improvement.	NR	NR	Planned: fasting, CT Unplanned: tube removal, tube loss, high GRV	Avoidable: fasting, tube issues Unavoidable: high GRV, CT	Procedural: fasting, CT, tube issues Intolerance: high GRV
Heyland et al. (6)	Canada	Multicenter prospective study	3,390 mechanically ventilated patients	Record underfeeding; supplement with PN.	74% missed 80% target; avg 61.2% calories, 57.6% protein.	NR	NR	Unplanned: diarrhea, vomiting, increased GRV	Unavoidable: diarrhea, vomiting, increased GRV	Intolerance: diarrhea, vomiting, increased GRV
Ramakrishnan et al. (33)	India	Prospective observational	*n* = 327 ICU patients	GRV > 500 mL threshold, prokinetics if >250 mL	Procedures 55.9% of ENI; GI 24.2%	NR	NR	Planned: procedures Unplanned: GI symptoms	Unavoidable: GI symptoms	Procedural: procedures Intolerance: GI symptoms
Ribeiro et al. (34)	Brazil	Prospective observational		Continuous EN (22 h/day), prokinetics	Adequacy 82.2%; tube removal main cause	NR	NR	Unplanned: tube removal, GI symptoms	Avoidable: tube removal Unavoidable: GI symptoms	Procedural: tube removal Intolerance: GI symptoms
Pasinato et al. (35)	Brazil	Prospective cohort study	*n* = 93 ICU patients	Implement EN protocols; assess adherence; support data-driven policy.	63% had early EN; 50% met goals by day 3; ENI from GI issues, instability.	NR	NR	Planned: surgery Unplanned: hemodynamic instability, GI issues	Unavoidable: hemodynamic instability, GI issues	Procedural: surgery Intolerance: GI issues
Passier et al. (36)	Australia	Retrospective	Trauma/surgical ICU	Shorten pre/post-procedure fasting, early EN resumption	ENI 7.9% of ICU time; 23% unnecessary; calorie/protein deficits 7.2%/7.7%	Periprocedural interruption of nutrition	NR	Planned: procedures Unplanned: tube issues	Avoidable: tube issues, unnecessary fasting	Procedural: procedures, tube issues
Uozumi et al. (37)	Japan	Retrospective	*n* = 100 ICU patients	ENI protocol, consensus adherence	ENI = 19% of feeding time; median 5.5 h/event	NR	NR	Planned: airway procedures Unplanned: GI symptoms, tube issues	Avoidable: tube issues	Procedural: airway procedures, tube issues Intolerance: GI symptoms
C. Observational studies (n = 20).										
Angotti et al. (38)	USA	Retrospective	*n* = 56 ICU patients	Continue feeding peri-tracheostomy	Tube feeding ↓ calorie deficit; no ↑ complications	NR	NR	Planned: anesthesia-related feeding interruption	Unavoidable: anesthesia-related feeding interruption	Procedural: anesthesia-related feeding interruption
Lee et al. (39)	Malaysia	Prospective	ICU patients on EN	Feed until ≤6 h pre-anesthesia, standard protocol, prokinetics, conduct regular training updates.	ENI 12.8% of nutrition days; ↓ calories (−1780 kcal) and protein (−100 g)	NR	NR	Planned: intubation/extubation, procedures Unplanned: GI intolerance	Avoidable: human factors, Unavoidable: GI intolerance	Procedural: intubation/extubation, procedures, human factors Intolerance: GI intolerance
Coutris et al. (40)	Canada	Retrospective	*n* = 27 adult burn	Compensation for predictable ENI	Predictable ENI: 74.5% (frequency), 81.6% (duration)	NR	NR	Planned: intraoperative Unplanned: tube removal, high GRV, GI intolerance	Unavoidable: intraoperative, High GRV	Procedural: intraoperative, tube removal Intolerance: high GRV, GI intolerance
Kıter et al. (41)	Turkey	Prospective	*n* = 80 ICU patients	Education, severe hemodynamic instability (MAP < 40 mmHg)	17.1% calorie deficit; airway management most common cause	NR	NR	Planned: airway procedures Unplanned: GI intolerance, tube dysfunction	Avoidable: tube dysfunction Unavoidable: GI intolerance,	Procedural: airway procedures Intolerance: GI intolerance
Morgan et al. (42)	USA	Retrospective observational	*n* = 56 trauma ICU patients	Multidisciplinary EN program	Received 67% prescribed calories	Temporary interruptions of tube feeding	NR	Planned: surgery, diagnostic procedures Unplanned: GI intolerance	Unavoidable: GI intolerance	Procedural: surgery, diagnostic procedures Intolerance: GI intolerance
Yeh et al. (43)	USA	Retrospective	Surgical ICU	Aggressive EN protocol (high protein, compensatory feeding)	↑ intake; ↓ late-stage infection	NR	NR	Unplanned: GI intolerance	Unavoidable: GI intolerance	Intolerance: GI intolerance
Peev et al. (44)	USA	Prospective observational	*n* = 94 surgical ICU	Tiered GRV processing, PEP uP scheme	ENI ↑ feeding inadequacy, LOS	Transient interruptions < 10 min not recorded	<10 min (excluded)	Planned: procedures Unplanned: high GRV	Unavoidable: high GRV	Procedural: procedures Intolerance: high GRV

ENI, enteral nutrition interruption; EN, enteral nutrition; ICU, intensive care unit; GRV, gastric residual volume; GI, gastrointestinal; LOS, length of stay; NR, not report; MAP, mean arterial pressure; PT, physical therapy; ECMO, extracorporeal membrane oxygenation; RRT, renal replacement therapy; ACS, abdominal compartment syndrome; HOB, head-of-bed.

## 3 Results

### 3.1 Characteristics of included studies

This scoping review included a total of 29 studies, covering ICU environments in various countries and regions such as North America, Europe, Asia, Oceania, and South America. The study subjects included critically ill patients in ICUs such as surgery, neurology, burns, trauma, respiratory failure, etc. The sample size ranged from 50 to 2000, and included adult patients aged 18 and above. The study designs included RCTs, quasi-experimental studies, observational studies, clinical guidelines, and evidence-based practice or quality improvement projects. The themes focused on the mechanism of ENI, its clinical impact, and precise nursing intervention measures. Tables 1A–C summarizes the basic characteristics of the 29 representative studies, including the authors, publication year, country, study design, sample characteristics, ENI definition, threshold, reasons for interruption, intervention measures, and main results. The studies covered different ICU subgroups to ensure the universality and specificity of the results.

### 3.2 Literature screening process

The literature screening followed the PRISMA-ScR guidelines and was accomplished through three stages of search. A total of 69,666 articles were retrieved, and after eliminating duplicates, 9,267 remained. After screening based on titles and abstracts, 108 articles were included for full-text evaluation, and finally, 29 studies were included. The screening process included: (1) initial search of PubMed and CINAHL for identifying keywords; (2) Comprehensive search of PubMed, Web Of Science, Cochrane Library, JBI, CINAHL, Embase, and Scopus; (3) Reference checking to supplement missing literature. The screening process was independently completed by two researchers, and disagreements were resolved through discussion or third-party adjudication. The detailed screening process can be found in the PRISMA-ScR flow diagram (Figure 1).

### 3.3 Variability in ENI definitions

The operational definition of ENI varied considerably across the included studies. In general, three definitional approaches were identified. First, some studies adopted frequency-based definitions, considering any interruption in EN delivery as an interruption event. Second, several studies used duration-based definitions, typically defining ENI as a cessation of enteral feeding lasting at least 1 h during continuous infusion. Third, some studies defined ENI based on specific clinical triggers, such as diagnostic or therapeutic procedures, gastrointestinal intolerance, tube-related complications, or hemodynamic instability. Given the substantial heterogeneity in ENI operational definitions and thresholds, we did not perform quantitative pooling of incidence or duration outcomes; instead, we mapped evidence descriptively and interpreted reported rates in relation to definitional approaches.

### 3.4 The reasons for the ENI

**FIGURE 2:**
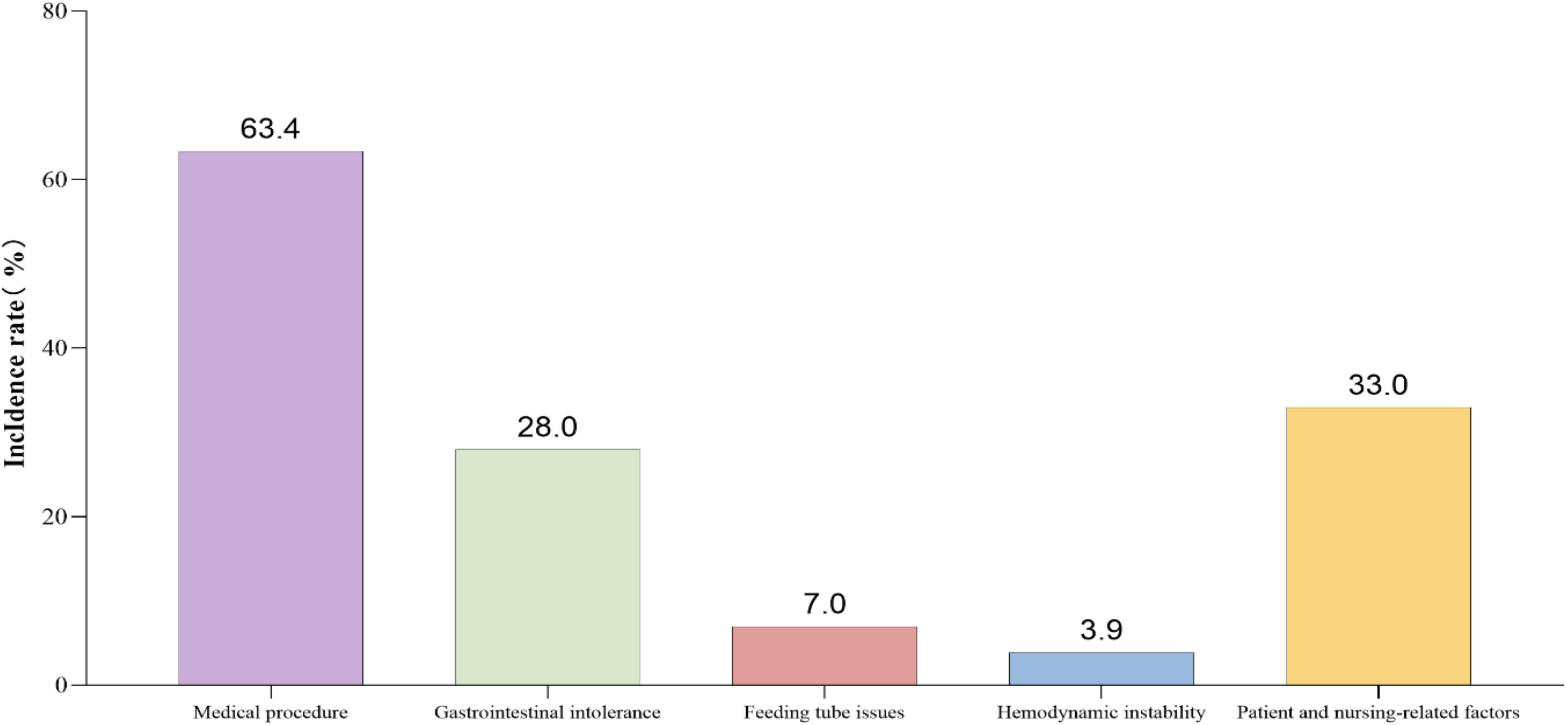
Causes and incidence of enteral nutrition interruption.

The causes of ENI can be classified into five major categories: diagnostic procedures, gastrointestinal intolerance, feeding tube problems, hemodynamic instability, and patient and nursing-related factors. The causes often interweave with each other, a single interruption event may report multiple contributing factors, these categories are not mutually exclusive, and the aggregated percentages may exceed 100% (17, 18, 45) (Figure 1). The interruption characteristics vary among different ICU subgroups. For instance, burn patients experience more frequent interruptions due to their high metabolic demands, while neurological patients often need to suspend feeding due to the risk of aspiration (46, 47). The distribution of reported ENI causes is summarized in Figure 2.

#### 3.4.1 Diagnostic procedures

The diagnostic procedures are the main cause of ENI, accounting for 63.4% of the incidents and 52.1% of the interruption duration (10). Common procedures include imaging examinations, bedside procedures, surgeries and endoscopic examinations. Among them, bedside procedures such as intubation and extubation account for approximately 38%, surgeries account for about 36%, and imaging examinations like CT and MRI and endoscopic examinations account for 30% and 10%, respectively. These procedures usually require patients to fast for 4–8 h (14, 45, 48, 49). For instance, routine fasting before radiological examinations leads to an average interruption of 6 h, and suspending feeding before tracheal intubation is a standard practice (14, 37). Among surgical ICU patients, the proportion of interruptions due to frequent surgeries is as high as 27%, while in neuro ICU patients, interruptions caused by imaging examinations are also quite common, with a proportion of approximately 10% (42, 50). Lu et al. (10) found that planned interruptions significantly reduced the time for nutritional infusion and exacerbated metabolic stress.

#### 3.4.2 Gastrointestinal irritation

The interruption events caused by gastrointestinal irritation accounted for 28%. The clinical manifestations mainly included increased gastric retention, diarrhea, vomiting, abdominal distension or intestinal obstruction. Among them, the incidence of gastric retention was approximately 17%, diarrhea was 8.4%, vomiting was 2.9%, and abdominal distension was 1.1% (16, 31, 39). The reasons include infection, shock, postoperative intestinal paralysis, sedative drugs, etc., which cause delayed gastric emptying and weakened intestinal peristalsis (51). When GRV is greater than or equal to 500 ml per 6 h or when intra-abdominal pressure is greater than 20 mmHg and accompanied by severe abdominal distension, feeding is usually suspended (52, 53). A prospective study showed that the median duration of interruption was 5.2 h, of which 3 h were due to handling high GRV (18). In burn patients, gastrointestinal intolerance is more common due to the high metabolic stress state; while in neuro-ICU patients, it is also prone to interruption due to the influence of sedative drugs (46, 50).

#### 3.4.3 Feeding tube issues

Feeding tube problems accounted for 7% of the interrupted events and 11.6% of the interrupted duration. Tube blockage and displacement accounted for 12% and 10% of the causes of ENI, respectively. Difficulties in catheter insertion accounted for 5.6% of the reasons for interruption. The average interruption lasted for 20 h and re-catheterization for the patients was required (14, 16, 32). Patients in the surgical ICU have a relatively high risk of catheter detachment after surgery, with an incidence rate of approximately 15%–20%; for burn patients, due to the need for frequent body position adjustments, the problem of catheter issues is more prominent, with an incidence rate of 10%–30% or even higher levels (42, 54).

#### 3.4.4 Hemodynamic instability

Hemodynamic instability accounted for 3.9% of the events and 2.6% of the interruption duration. For instance, in cases of shock or when high-dose vasopressors are required and the mean arterial pressure is below 50 mmHg, the nutritional infusion needs to be suspended. After the patient’s hemodynamics stabilize, it can be restarted at a low speed within 24–48 h (3). The proportion of patients in the trauma and surgical ICU who experienced interruption in nutritional infusion due to shock was relatively high, approximately 4% (45).

#### 3.4.5 Factors related to patients and nursing

Patient factors: When upper gastrointestinal bleeding or intestinal obstruction occurs, nutritional infusion should be suspended. For patients with low-risk bleeding, nutritional infusion can be resumed within 24–48 h (1, 10). Neurological patients have a higher risk of aspiration, and the interruption of nutritional infusion is more frequent, with an interruption rate of up to 30% (55).

Nursing factors: Interruptions in nutritional infusion caused by nursing-related factors account for approximately 33% (11). Some nurses may misjudge the indications for suspension. For instance, they might consider a Gastric Residual Volume (GRV) of ≥200 mL as the basis for suspension, instead of the recommended ≥500 mL/6 h as per the guidelines. The insufficient coverage of systematic training has led to the medical staff having a poor understanding of the definition, causes, and consequences of ENI. Under high workloads, rescue tasks, evaluations, and paperwork can easily occupy feeding time, prolonging the interruption period by 6–8 h. Routine operations such as position adjustment, suctioning, and preparation for examinations, if lacking standardized procedures and quality management tools specific to ENI, can also become avoidable sources of interruption (20). Insufficient communication among nurses, doctors and nutritionists will further aggravate the interruption of nutritional infusion, and this phenomenon is particularly prominent in neurology ICUs and burn ICUs (46, 56).

### 3.5 Clinical impact and mechanism

Enteral nutrition interruption has been associated with inadequate energy and protein delivery and may contribute to higher risks of malnutrition, infection, and prolonged hospitalization (12). An observational study revealed that the incidence of nutritional deficiency among ENI patients rose to 54.0%, significantly higher than 15.0% among patients without interruptions; the ICU mortality rate increased to 46.0%, while it was only 21.7% for those without interruptions; moreover, the interruption also led to an extension of hospital stay and an increase in medical expenses (12, 13). A prospective study reported that patients experiencing ≥3 cumulative interruptions had a higher risk of mortality (adjusted odds ratio 6.73) and shorter survival time compared with those with fewer interruptions (14). There are significant differences in the impact of different ICU subgroups. Burn patients have a more severe case of malnutrition due to their high metabolic demands, with approximately 50%–60% of cases; while neurology patients have difficulty removing the ventilator due to muscle weakness (55, 57).

Intestinal barrier damage: Nutritional deprivation may contribute to mucosal atrophy, villus shortening, reduced intestinal gland activity, decreased mucus secretion, increased intestinal permeability, and reduced expression of tight junction proteins. These alterations may facilitate bacterial translocation and endotoxin entry, which could potentially promote systemic inflammation and increase the risk of multiple organ dysfunction syndrome (58). Burn patients have a higher rate of catabolic metabolism, which leads to more significant damage to the intestinal barrier and makes them more prone to bacterial translocation (40).

Intestinal microecological imbalance: Critical illness and malnutrition may be associated with reductions in beneficial bacteria and increases in potentially pathogenic bacteria. In addition, antibiotic use may further reduce microbial diversity, which could potentially increase the risk of infection (58). Among the population using antibiotics, ENI may lead to an increase in the infection rate (58).

Overall effects: A high catabolic state in critically ill patients may be associated with significant skeletal muscle loss and the development of ICU-acquired muscle weakness, which has been reported in approximately 40% of patients. This condition may contribute to prolonged mechanical ventilation, delayed wound healing, and increased oxidative stress (59, 60). Patients whose energy intake was less than 65% of the target value had a significantly higher mortality rate, with an OR of approximately 4.97, 95% CI 1.44–17.07; patients with energy intake of 65% or more had significantly lower mortality rates at 30, 60, and 90 days (61). Patients with neurological disorders experience delayed functional recovery due to muscle weakness (55).

### 3.6 Precise nursing intervention strategy

Precise nursing intervention aims to reduce ENI through gastrointestinal management, technical adjustments, monitoring improvements, risk assessment, and team collaboration. Nurses play a leading role in the implementation and monitoring process. The specific strategies are as follows:

#### 3.6.1 Optimization of feeding process and care pathway

Under the application of the standardized feeding pathway such as the PEP uP scheme, the rate of patients achieving energy targets increased from 26.9% to 57.0%, and the rate of protein targets increased from 18.6% to 57.4%. No increase in the risk of hyperglycemia was observed. Under a more adequate nutritional condition, no significant increase in vomiting, gastrointestinal intolerance or hyperglycemic events was noted. Even some data suggested that the rate of hyperglycemic events decreased (62). The capacity feeding method increased energy by 15.4% and protein by 22.1% compared to the rapid feeding method, and shortened the ICU stay time by 0.8 days (63). The nurse-led standardized EN pathways, such as PEP uP, volume-based feeding protocols, and the early EN best practice guidelines, generally require nurses to check the actual intake amount every shift, actively adjust the pump speed to catch up when there is an interruption, and communicate with the surgical or examination teams to shorten unnecessary interruptions. Multiple studies have confirmed that these pathways can significantly increase the implementation rate of early EN and advance the EN initiation time by approximately 0.5–1 day (24, 64).

#### 3.6.2 Adjusting feeding methods

Compared with continuous feeding, intermittent feeding increases the risk of diarrhea by approximately 66%, the incidence of abdominal distension is about 2.3 times higher, the length of ICU stay is prolonged by approximately 1 day, and there is no difference in the occurrence of aspiration pneumonia or mortality rate (65). Continuous feeding can prevent fluctuations in the feeding contents and is suitable for patients with frequent interruptions. ICU patients can benefit from it (66). The nurse is responsible for monitoring the pump speed at night to ensure continuity, especially in the neuro ICU where it can significantly reduce the risk of aspiration (67).

#### 3.6.3 Improvement in gastric residual volume monitoring

For most adult patients with critical conditions, routine monitoring of gastric residual volume is not recommended, as it is an indirect and unreliable indicator, and is susceptible to influences such as the aspiration technique, tube gauge, and the patient’s body position (68). Over-reliance on gastric residual volume as the basis for interruption EN may lead to unnecessary ENI, thereby increasing the risk of insufficient calorie supply (69). Therefore, nursing practice should prioritize maintaining EN continuity and avoid unnecessary ENI solely driven by GRV values. When intolerance is suspected, a symptom-based and multimodal assessment should be adopted, including evaluation for vomiting and abdominal distension, as well as the use of bedside ultrasound when available (3, 23, 70). Consistent with guideline-based approaches, EN should be temporarily withheld only when GRV is markedly elevated to 500 mL or higher in combination with clinical signs suggesting intolerance, rather than as a routine scheduled measurement (3). Nurses acquired ultrasound monitoring skills through training, reducing unnecessary ENI. This measure was particularly effective in the burn ICU where the incidence of GRV was high (47). Overall, minimizing avoidable GRV driven interruptions represents a practical strategy to reduce caloric under-delivery and improve nutrition delivery in critically ill patients.

#### 3.6.4 Reducing planned interruptions

To reduce planned ENI, optimization of feeding modality and fasting practices is essential. Current evidence indicates that intermittent feeding is clinically comparable to continuous feeding in mechanically ventilated critically ill adults, with no significant differences in mortality, gastrointestinal intolerance, pneumonia, or ICU length of stay (71). This supports selective use of intermittent or continuous feeding to improve scheduling flexibility and compensate for predictable ENI. Procedure related fasting is another modifiable contributor to avoidable interruption. A large multicenter randomized trial showed that continuing EN until extubation was non inferior to a 6 h pre extubation fasting strategy in terms of extubation failure and pneumonia, while routine fasting before airway procedures reduced caloric delivery without clear safety benefit (72). Collectively, these findings suggest that prolonged routine fasting in patients receiving EN, particularly those with protected airways, is often unnecessary (72). Individualized fasting protocols combined with flexible infusion strategies may therefore help minimize avoidable ENI and cumulative caloric deficits in critically ill adults.

#### 3.6.5 Supplementary measures

Stimulant drugs: Erythromycin, Metoclopramide can reduce patient’s intolerance and increase the rate of achieving nutritional standards. Nurses monitor the side effects of the drugs to ensure the safety of the dosage (73).

Dietary fiber and probiotics: In critically ill adults, the potential role of fiber varies according to fiber composition and patient selection. Fermentable soluble fiber may be considered in hemodynamically stable patients with persistent diarrhea after other reversible causes have been addressed, typically administered at 10–20 grams per day in divided doses. Commercial mixed fiber formulations containing both soluble and insoluble components may be considered in selected patients with constipation. However, routine fiber supplementation is not generally recommended in the ICU and should be avoided in patients at high risk of bowel ischemia or severe gastrointestinal dysmotility. In patients with persistent diarrhea and suspected malabsorption or inadequate response to fiber, small peptide based enteral formulations may be considered. With respect to adjunctive therapies, current evidence does not support routine probiotic use in the general critically ill population. Although studied probiotic strains appear safe in many ICU settings, consistent reductions in infection related outcomes, ventilator associated pneumonia, or *Clostridioides difficile* associated diarrhea have not been clearly demonstrated. Probiotics, if used, should therefore be limited to carefully selected medical or surgical populations in which specific strains have documented safety and clinical benefit (3, 74).

Jejunum feeding: For patients with persistent gastric intolerance or high aspiration risk, post-pyloric feeding may be considered as part of an interruption-mitigation strategy when feasible. Nursing practice should emphasize tube-position verification, securement, and timely troubleshooting to reduce dislodgement and support feeding continuity (75).

#### 3.6.6 Strengthen monitoring and collaboration

The dedicated nurse summarizes the actual intake of patients and the preset energy targets on a daily basis, such as 80% of the target intake. Combined with the information system, automatic alerts are set. When the actual intake is lower than the target, the nurse team can quickly identify the problem and take necessary remedial measures, thereby effectively reducing the occurrence of feeding interruptions (19). When dealing with ENI, the nurses evaluated their understanding and coping abilities regarding ENI by using the Knowledge-Attitude-Practice Scale, and enhanced their theoretical and practical knowledge through specialized training (19). Studies have shown that after training, nurses’ knowledge scores in ENI management have significantly improved, and the interruption rate has also decreased accordingly (20). Furthermore, the nutrition support team worked closely with the nurses to jointly assess complex cases and develop personalized nutrition plans, thereby significantly improving the actual intake of patients (76). Further examination reveals that the nurse-led VBF protocol has been widely implemented. By strengthening the daily intake goal management and the verification of feeding logs, it ensures the achievement of the goals and reduces process omissions (63). In the mixed-type ICU, the multidisciplinary collaboration and nurse-led process optimization significantly reduced the frequency of ENI while greatly increasing the rate of energy compliance (26).

## 4 Discussion

This scoping review comprehensively examined the mechanisms, clinical impacts, and precise nursing intervention measures of ENI interruption in critically ill patients in the ICU. It provided evidence-based support for optimizing ICU nursing practices. The study found that ENI is a major obstacle to nutritional support in the ICU, with an average occurrence rate of 48.3%. Each patient experiences an average of 2–3 interruptions, with a cumulative interruption time of approximately 33.8 h (10). The reported incidence of ENI ranged widely from 4.7% to 100%, which is likely related to heterogeneous operational definitions and methodological differences across studies. The main reasons include diagnostic and therapeutic procedures, accounting for 63.4%; gastrointestinal intolerance, accounting for 19.2%; feeding pipeline issues, accounting for 9.3%; hemodynamic instability, accounting for 3.9%; and nursing-related factors, accounting for 33% (10, 11). ENI has been associated with suboptimal nutritional delivery and a higher risk of malnutrition, with reported incidence ranging from 38% to 78%. It may also be linked to intestinal barrier dysfunction, alterations in gut microbiota, and systemic metabolic disturbances, which could potentially increase the risk of infection and stress-related complications. Furthermore, some studies have suggested that ENI may be related to a longer hospital stay, with increases of approximately 3–5 days (12, 13, 58). Furthermore, one study reported that patients experiencing three or more interruptions had a higher risk of mortality (adjusted odds ratio approximately 6.7) (14). Nurse-led targeted nursing strategies, such as standardized feeding pathways, continuous infusion, monitoring of gastric residual volumes, perioperative optimization, and adjunctive measures including prokinetic agents, probiotics, and jejunal feeding, may help reduce the frequency and duration of feeding interruptions and improve energy and protein delivery. The energy compliance rate increased from 26.9% to 57.0%, and the protein compliance rate rose from 18.6% to 57.4% (10, 22, 62). This study highlights the core role of nurses in the management of EN, filling the knowledge gap in the existing literature from a nursing perspective and providing practical guidance for ICU nurses.

The findings of this review are consistent with existing research, but offer a more systematic analysis from the perspective of nursing. The systematic review by Lu et al. (10) reported ENI incidence ranging from 4.7% to 100%, with an average of 48.3%, consistent with this study, emphasizing the dominant role of diagnostic and therapeutic procedures and gastrointestinal intolerance. Salciute-Simene et al. (31) and Onuk et al. (14) confirmed that discontinuation is associated with higher rates of malnutrition and mortality, at 54.0% vs. 15.0% and 46.0% vs. 21.7%, underscoring the potential clinical relevance of ENI and the importance of minimizing avoidable interruptions. This review further analyzes that nursing-related factors such as knowledge gaps and process deficiencies account for approximately 33% of nutritional infusion interruptions, thereby highlighting the critical role nurses play in reducing such interruptions (11, 20). For instance, some nurses wrongly judged the indications for GRV suspension, mistakenly setting the threshold at more than 200 mL instead of the 500 mL recommended by the guidelines, resulting in unnecessary interruptions of feeding (1, 20). Carrying out systematic training and quality improvement programs for nursing staff can significantly reduce the number of days of inadequate feeding and ENI caused by nursing-related factors, and increase the proportion of achieving the set energy targets (77). Compared with the relatively simple behavioral interventions in other nursing scenarios, the management of ENI in the ICU relies more on continuous observation of the patient’s condition and high-level collaboration among the team. The condition of critically ill patients changes rapidly, and the treatment measures are intensive, which requires the nutritional infusion to remain stable in a dynamic environment (78, 79). The evidence-based review indicates that within a framework involving nutritionists, nurses, dietitians, and clinical pharmacists, by integrating nurse-led feeding strategies, standardized tools, and information-based monitoring, it is possible to better maintain the consistency of nutritional supply (79). Based on this, the standardized protocols such as PEP uP and VBF feeding were developed. By setting a 24-h volume target and allowing nurses to dynamically adjust the infusion rate according to interruptions, these protocols significantly improved the supply of energy and protein. In a before-and-after comparative study in an orthopedic trauma ICU, the application of such strategies increased the proportion of ≥80% energy targets from 26.9% to 57.0% (62). The ESPEN guidelines recommend EN as the preferred mode of nutrition (1). This scoping further clarifies how nurses can overcome the obstacles of interruption through precise care, providing specific strategies for the clinical implementation of the guidelines.

The effectiveness of precision nursing interventions was particularly evident across different ICU subgroups. Standardized feeding pathways such as the PEP uP protocol substantially improved energy and protein delivery, increasing attainment rates from 26.9% and 18.6% to 57.0% and 57.4%, respectively (62, 63). Volume-based feeding strategies further improved nutritional delivery and reduced interruption events in surgical ICU populations (63). Continuous infusion approaches were associated with improved gastrointestinal tolerance and were particularly beneficial for neuro-ICU patients with a high aspiration risk (28, 65). Similarly, reducing reliance on routine gastric residual volume monitoring did not increase mortality or pneumonia and helped avoid unnecessary feeding interruptions (47, 80, 81). Optimization of perioperative fasting practices and coordinated scheduling of diagnostic procedures also supported continuity of nutritional support in surgical ICU settings (36, 82). In addition, strategies targeting feeding intolerance, including prokinetic therapy, probiotics, and post-pyloric feeding, were associated with improved nutritional target attainment in selected patient groups (73, 75, 83). These findings highlight the critical role of nurse-led monitoring and multidisciplinary collaboration in reducing avoidable interruptions and maintaining EN delivery. Practices such as feeding record verification, continuous evaluation of interruption causes, and coordinated team-based management were associated with improved continuity of EN and higher rates of nutritional target achievement (26). To translate these findings into practical ICU nutrition management, we propose an operational framework integrating risk stratification, structured monitoring, and targeted strategies to reduce ENI.

Risk stratification: At the initiation of EN and during daily reassessment, nurses should evaluate interruption risk across key domains including nutrition risk, aspiration risk, anticipated procedures, gastrointestinal intolerance, and hemodynamic instability (1–3, 14, 17, 18). These factors were consistently identified as major contributors to ENI across the included studies. Patients with higher risk profiles may benefit from intensified monitoring and proactive interruption-prevention planning (19, 20, 26).

Triggers for action and monitoring: Maintaining feeding continuity requires structured nurse-led monitoring of delivered versus prescribed nutrition, cumulative interruption duration, interruption frequency, and documented causes of ENI (6, 14, 18, 26, 29). Particular attention should be given to procedure-related, intolerance-related, and human factors-related interruptions, which represented the predominant factors across studies (14, 17, 18, 29). Corrective actions may be warranted when interruptions are prolonged, recurrent, or associated with failure to achieve daily nutrition targets (19, 20, 26, 63).

Pragmatic ENI prevention strategies: Several practical approaches may help reduce interruption burden in routine ICU care. These include minimizing unnecessary pre-procedure fasting and ensuring timely resumption of feeding after procedures (17, 29, 36, 38). Feeding intolerance should be assessed primarily based on clinical symptoms rather than modest gastric residual volumes alone (3, 18, 23, 33). In patients with persistent intolerance or high aspiration risk, prokinetic therapy and post-pyloric feeding may be considered (33, 39, 75). Strategies such as continuous infusion and volume-based feeding may further support nutrition delivery despite predictable interruptions (25, 28, 62, 63).

Because tube malfunction or displacement was also a common cause of ENI, routine tube care, troubleshooting protocols, and clear documentation of interruption events remain essential components of nursing management (14, 32, 37, 40). In addition, feeding-log verification and multidisciplinary review of interruption causes may help improve the continuity of EN delivery (19, 20, 26, 63).

### 4.1 Limitations and future research directions

This scoping review comprehensively examined the mechanisms, impacts, and precise nursing intervention measures for ENI in critically ill patients in the ICU, providing evidence-based guidance for optimizing nursing practices. However, it has the following limitations that need to be interpreted with caution. The definitions and assessment criteria for ENI, such as frequency, duration, or GRV threshold, vary significantly across different studies. For instance, studies that define ENI by “interruption > 4 h” have a higher incidence rate than those defined by frequency, potentially overestimating the clinical impact. Most studies are concentrated in North America, Europe, and Asia, with insufficient data from Africa and South America. For ICUs lacking night-time nursing support and resources, implementing continuous infusion and other intervention measures faces significant difficulties. The research lacks studies on subgroup-specific issues such as high metabolic needs in burn patients and high aspiration risk in neurological patients, and there is limited evidence for personalized interventions. Evidence for some adjunctive measures, including probiotics and dietary fiber, remains inconsistent across studies, partly due to heterogeneity in study designs, populations, and outcome definitions. Several observational studies were limited by retrospective designs and small sample sizes, which may affect the stability of reported associations. Continuous infusion and bedside ultrasound monitoring require resource support and training. In some regions, medical staff have concerns about discontinuing routine GRV monitoring, which limits the promotion and application of these measures. Additionally, positive results may be prioritized, leading to publication bias risks.

To overcome these limitations and optimize EN management, future research can be conducted in the following directions. Standardized evaluation criteria for ENI should be established, taking into account frequency, duration and the proportion of nutritional deficiencies, to enhance data consistency. Studies in regions such as Africa and South America should be increased to explore adaptive strategies in resource-limited environments. Personalized feeding protocols should be developed for patients with burns and neurological disorders, and their effectiveness should be verified through multi-center RCTs. Large-scale RCTs should be carried out to verify the effects of probiotics, dietary fibers, etc., to reduce bias. Gastric motility monitoring devices, ENI risk prediction models and intelligent feeding pumps should be developed to dynamically optimize the intervention. Pilot studies have shown that the intelligent pump can reduce interruptions by 2 h. A virtual reality training module should be developed, through simulating complex clinical cases such as aspiration prevention, to effectively reduce feeding interruption events caused by insufficient knowledge. Cross-regional nutrition support teams should be established to optimize feeding plans for complex cases, achieving 80% of nutritional goals. The implementation of scientific methods should be adopted to solve the barriers to intervention promotion in resource-limited ICUs, ensuring the sustainability of precise care. These directions will enhance intervention effects and improve the nutritional outcomes and quality of life of ICU patients.

## 5 Conclusion

This scoping review examined the mechanisms, impacts, and nurse-led management strategies for ENI in ICU patients. ENI occurred frequently, with an incidence of approximately 48.3%, most commonly related to diagnostic procedures, gastrointestinal intolerance, and device-related issues. These interruptions were associated with inadequate nutritional delivery and adverse clinical outcomes, particularly in high-risk subgroups such as burn and neurological patients. Evidence from the included studies suggests that nurse-led precision interventions, including standardized feeding pathways, optimized fasting management, symptom-based intolerance assessment, and structured monitoring of nutrition delivery, may help reduce avoidable interruptions and improve energy and protein attainment. However, the effects of these interventions on broader clinical outcomes such as infection, length of stay, mortality, and functional recovery remain uncertain and require confirmation in higher-quality studies. Future research should prioritize standardized ENI definitions, subgroup-specific investigation, and integrated monitoring strategies to further improve nutritional support and optimize EN management in critically ill patients.

## Supplementary material

Supplementary Material Full search strategies used in this scoping review.
