# Supplementary material for: Interruptions of enteral nutrition in intensive care units: mechanisms, clinical impacts, and precision nursing interventions–a scoping review
Source: Front Nutr. 2026 May 28;13:1768983. doi: 10.3389/fnut.2026.1768983 (PMC13253397; doi:10.3389/fnut.2026.1768983)
Supplement: Supplementary file 1 [file Supplementary_file_1.docx]

This review employs a three-stage search strategy, retrieving literature from PubMed, Web of Science, Cochrane Library, JBI, CINAHL, Embase, and Scopus, covering publications from the inception of each database up to April 2025. The following details the search strings, retrieval settings, and number of records for each database. The search language was limited to Chinese and English, with search fields including titles, abstracts, and keywords, and no restrictions on publication year. All retrieval results were imported into EndNote software for deduplication, with detailed records maintained to ensure reproducibility.

1.Pubmed

Search string：

("enteral feed" OR "Enteral nutrition" OR "gastric tube feeding" OR "gastrostomy nutrition" OR "intestinal feeding" OR "intestinal nutrition" OR "jejunostomy feed*" OR "jejunostomy nutrition" OR "nasal feeding" OR "naso intestinal tube feeding" OR "nasogastric feed*" OR "nasointestinal tube feeding" OR "tube feeding") And ("cessat*" OR "discontinu*" OR "disrupt*" OR "feeding break*" OR "interrupt*" OR "nutrition break*" OR "stop*" OR "withhold*")

MeSH terms：("Enteral Nutrition"[Mesh])

Settings: Language（English）

Search field（All Fields），no year restriction

Number of records: 16,616

2.Web Of Science

Search string：

(ALL=("enteral feed" OR "Enteral nutrition" OR "gastric tube feeding" OR "gastrostomy nutrition" OR "intestinal feeding" OR "intestinal nutrition" OR "jejunostomy feed*" OR "jejunostomy nutrition" OR "nasal feeding" OR "naso intestinal tube feeding" OR "nasogastric feed*" OR "nasointestinal tube feeding" OR "tube feeding")) AND (ALL=("cessat*" OR "discontinu*" OR "disrupt*" OR "feeding break*" OR "interrupt*" OR "nutrition break*" OR "stop*" OR "withhold*"))

Settings: Language（English）

Search field（All Fields），no year restriction

Number of records: 11,125

3.Cochrane Library

Search string：

("enteral feed" OR "Enteral nutrition" OR "gastric tube feeding" OR "gastrostomy nutrition" OR "intestinal feeding" OR "intestinal nutrition" OR "jejunostomy feed*" OR "jejunostomy nutrition" OR "nasal feeding" OR "naso intestinal tube feeding" OR "nasogastric feed*" OR "nasointestinal tube feeding" OR "tube feeding") And ("cessat*" OR "discontinu*" OR "disrupt*" OR "feeding break*" OR "interrupt*" OR "nutrition break*" OR "stop*" OR "withhold*")

Settings: Language (English)

Search field（All Text），no year restriction

Number of records: 3,370

4.JBI

Search string：

("enteral feed" OR "Enteral nutrition" OR "gastric tube feeding" OR "gastrostomy nutrition" OR "intestinal feeding" OR "intestinal nutrition" OR "jejunostomy feed*" OR "jejunostomy nutrition" OR "nasal feeding" OR "naso intestinal tube feeding" OR "nasogastric feed*" OR "nasointestinal tube feeding" OR "tube feeding") And ("cessat*" OR "discontinu*" OR "disrupt*" OR "feeding break*" OR "interrupt*" OR "nutrition break*" OR "stop*" OR "withhold*")

Settings: Language (English)

Search field（Full Text），no year restriction

Number of records: 200

5.CINAHL

Search string：

("enteral feed" OR "Enteral nutrition" OR "gastric tube feeding" OR "gastrostomy nutrition" OR "intestinal feeding" OR "intestinal nutrition" OR "jejunostomy feed*" OR "jejunostomy nutrition" OR "nasal feeding" OR "naso intestinal tube feeding" OR "nasogastric feed*" OR "nasointestinal tube feeding" OR "tube feeding") And ("cessat*" OR "discontinu*" OR "disrupt*" OR "feeding break*" OR "interrupt*" OR "nutrition break*" OR "stop*" OR "withhold*")

Settings: Language (English)

Search field（TX All Text）、no year restriction

Number of records: 1,533

6.Embase

Search string：

("enteral feed" OR "Enteral nutrition" OR "gastric tube feeding" OR "gastrostomy nutrition" OR "intestinal feeding" OR "intestinal nutrition" OR "jejunostomy feed*" OR "jejunostomy nutrition" OR "nasal feeding" OR "naso intestinal tube feeding" OR "nasogastric feed*" OR "nasointestinal tube feeding" OR "tube feeding") And ("cessat*" OR "discontinu*" OR "disrupt*" OR "feeding break*" OR "interrupt*" OR "nutrition break*" OR "stop*" OR "withhold*")

Settings: Language (English)

Search field（Broad search），no year restriction

Number of records: 26,747

7.Scopus

Search string:：

("enteral feed" OR "Enteral nutrition" OR "gastric tube feeding" OR "gastrostomy nutrition" OR "intestinal feeding" OR "intestinal nutrition" OR "jejunostomy feed*" OR "jejunostomy nutrition" OR "nasal feeding" OR "naso intestinal tube feeding" OR "nasogastric feed*" OR "nasointestinal tube feeding" OR "tube feeding") And ("cessat*" OR "discontinu*" OR "disrupt*" OR "feeding break*" OR "interrupt*" OR "nutrition break*" OR "stop*" OR "withhold*")

Settings: Language (English)

Search fields (TITLE-ABS-KEY Title Abstract Keywords)，no year restriction

Number of records: 10,075

Total

Total number of records retrieved: 69,666.(PubMed: 16,616, Web of Science: 11,125, Cochrane Library 3,370, JBI 200, CINAHL 1,533, Embase 26,747, Scopus 10,075, with some documents duplicated across multiple databases).

After deduplication in EndNote:9,267 articles.
