# Supplementary material for: Interruptions of enteral nutrition in intensive care units: mechanisms, clinical impacts, and precision nursing interventions–a scoping review
Source: Front Nutr. 2026 May 28;13:1768983. doi: 10.3389/fnut.2026.1768983 (PMC13253397; doi:10.3389/fnut.2026.1768983)
Supplement: Supplementary Material — Full search strategies used in this scoping review. [file Table_1.docx]

**Preferred Reporting Items for Systematic reviews and Meta-Analyses extension for Scoping Reviews (PRISMA-ScR) Checklist**

| **SECTION** | **ITEM** | **PRISMA-ScR CHECKLIST ITEM** | **REPORTED ON PAGE #** |
| --- | --- | --- | --- |
| **TITLE** | | | |
| Title | 1 | Identify the report as a scoping review. | Title page — “Interruptions of Enteral Nutrition in Intensive Care Units: Mechanisms, Clinical Impacts, and Precision Nursing Interventions—A Scoping Review” |
| **ABSTRACT** | | | |
| Structured summary | 2 | Provide a structured summary that includes (as applicable): background, objectives, eligibility criteria, sources of evidence, charting methods, results, and conclusions that relate to the review questions and objectives. | Abstract — structured (Background, Objective, Methods, Results, Conclusions). |
| **INTRODUCTION** | | | |
| Rationale | 3 | Describe the rationale for the review in the context of what is already known. Explain why the review questions/objectives lend themselves to a scoping review approach. | Although individual studies have explored specific aspects of ENI, the evidence remains fragmented across different study designs and clinical contexts. Because the literature includes heterogeneous evidence types such as observational studies, clinical guidelines, and implementation projects, a scoping review approach is appropriate for mapping the breadth of available evidence and identifying knowledge gaps. |
| Objectives | 4 | Provide an explicit statement of the questions and objectives being addressed with reference to their key elements (e.g., population or participants, concepts, and context) or other relevant key elements used to conceptualize the review questions and/or objectives. | This scoping review aimed to map the existing evidence regarding enteral nutrition interruption in ICU patients. Specifically, the review addressed the following objectives: (1) to identify the reported mechanisms and causes of ENI in critically ill patients; (2) to examine clinical outcomes associated with ENI, including nutritional inadequacy and patient prognosis; and (3) to summarize nurse‑led precision nursing interventions that may reduce interruptions and improve enteral nutrition delivery in ICU practice. |
| **METHODS** | | | |
| Protocol and registration | 5 | Indicate whether a review protocol exists; state if and where it can be accessed (e.g., a Web address); and if available, provide registration information, including the registration number. | This scoping review followed the Joanna Briggs Institute methodology and the Arksey–O’Malley framework and was reported according to PRISMA-ScR guidelines. No formal protocol was registered. |
| Eligibility criteria | 6 | Specify characteristics of the sources of evidence used as eligibility criteria (e.g., years considered, language, and publication status), and provide a rationale. | Eligible studies were English-language peer-reviewed publications involving adult ICU patients that examined mechanisms, clinical impacts, or management of enteral nutrition interruption. Randomized trials, observational studies, clinical guidelines, and quality improvement projects published up to April 2025 were included. Studies conducted outside ICU settings or non-empirical publication types were excluded. |
| Information sources* | 7 | Describe all information sources in the search (e.g., databases with dates of coverage and contact with authors to identify additional sources), as well as the date the most recent search was executed. | Searches were conducted in PubMed, Web of Science, Cochrane Library, JBI Database, CINAHL, Embase, and Scopus from database inception to April 2025. Reference lists of included studies were also screened. |
| Search | 8 | Present the full electronic search strategy for at least 1 database, including any limits used, such that it could be repeated. | Methods 2.3— Search strategy  "enteral feed" OR "enteral nutrition" OR "gastric tube feeding" OR "gastrostomy nutrition" OR "intestinal feeding" OR "jejunal feeding" OR "nasogastric feeding" OR "tube feeding") AND ("cessation" OR "discontinue*" OR "interrupt*" OR "feeding break*" OR "withhold*"  Search field（All Fields），no year restriction |
| Selection of sources of evidence† | 9 | State the process for selecting sources of evidence (i.e., screening and eligibility) included in the scoping review. | Records were imported into EndNote and duplicates removed. Two reviewers independently screened titles/abstracts and assessed full texts using predefined criteria, with disagreements resolved by consensus or a third reviewer. |
| Data charting process‡ | 10 | Describe the methods of charting data from the included sources of evidence (e.g., calibrated forms or forms that have been tested by the team before their use, and whether data charting was done independently or in duplicate) and any processes for obtaining and confirming data from investigators. | Two reviewers independently extracted data using a pilot-tested Excel charting form. Extracted data were cross-checked and discrepancies resolved through discussion. |
| Data items | 11 | List and define all variables for which data were sought and any assumptions and simplifications made. | Extracted variables included study characteristics, ENI definitions and thresholds, causes of interruption, clinical outcomes, and nurse-led intervention strategies. Causes of ENI were grouped into thematic categories due to study heterogeneity. |
| Critical appraisal of individual sources of evidence§ | 12 | If done, provide a rationale for conducting a critical appraisal of included sources of evidence; describe the methods used and how this information was used in any data synthesis (if appropriate). | Methodological appraisal was conducted using appropriate Joanna Briggs Institute tools according to study design. Appraisal results informed interpretation but were not used to exclude studies. |
| Synthesis of results | 13 | Describe the methods of handling and summarizing the data that were charted. | Charted data were summarized using narrative synthesis and organized into thematic categories corresponding to the review questions, including ENI mechanisms, clinical impacts, and nurse-led interventions. |
| **RESULTS** | | | |
| Selection of sources of evidence | 14 | Give numbers of sources of evidence screened, assessed for eligibility, and included in the review, with reasons for exclusions at each stage, ideally using a flow diagram. | A total of 69,666 records were identified. After removing duplicates, 9,267 records were screened, 108 full texts were assessed, and 29 studies were included. The selection process is presented in the PRISMA-ScR flow diagram. |
| Characteristics of sources of evidence | 15 | For each source of evidence, present characteristics for which data were charted and provide the citations. | Characteristics of included studies are presented in Table 1A–C and include author, year, country, study design, ICU population, ENI definitions, causes of interruption, interventions, and outcomes. |
| Critical appraisal within sources of evidence | 16 | If done, present data on critical appraisal of included sources of evidence (see item 12). | Methodological appraisal indicated heterogeneous study designs, including guidelines, interventional studies, and observational studies. These differences were considered when interpreting the findings. |
| Results of individual sources of evidence | 17 | For each included source of evidence, present the relevant data that were charted that relate to the review questions and objectives. | Data extracted from each included study included ENI definitions, causes of interruption, intervention strategies, and reported clinical outcomes relevant to the review objectives. |
| Synthesis of results | 18 | Summarize and/or present the charting results as they relate to the review questions and objectives. | Results were synthesized narratively and organized into three domains: mechanisms of ENI, associated clinical impacts, and nurse-led strategies to reduce interruptions and improve nutritional delivery. |
| **DISCUSSION** | | | |
| Summary of evidence | 19 | Summarize the main results (including an overview of concepts, themes, and types of evidence available), link to the review questions and objectives, and consider the relevance to key groups. | ENI commonly occurs in ICU practice and is frequently associated with diagnostic procedures, gastrointestinal intolerance, and device-related factors. Nurse-led interventions may improve continuity of enteral nutrition delivery and nutritional target attainment. |
| Limitations | 20 | Discuss the limitations of the scoping review process. | Variations in ENI definitions, heterogeneous study designs, and limited evidence from resource-limited settings may affect comparability and generalizability of findings. |
| Conclusions | 21 | Provide a general interpretation of the results with respect to the review questions and objectives, as well as potential implications and/or next steps. | ENI remains a common challenge in ICU nutrition support. Nurse-led strategies may improve nutritional delivery, but standardized ENI definitions and higher-quality studies are needed. |
| **FUNDING** | | | |
| Funding | 22 | Describe sources of funding for the included sources of evidence, as well as sources of funding for the scoping review. Describe the role of the funders of the scoping review. | This review was supported by the Research Development Fund Project of the Affiliated Teaching Hospital of Shandong Second Medical University (2025FYG002), the Hospital-level Nursing Education Project (2025HLB001), and the Weifang Key Laboratory for Clinical Nursing Research. Funding information for individual included studies was reported when available. |

JBI = Joanna Briggs Institute; PRISMA-ScR = Preferred Reporting Items for Systematic reviews and Meta-Analyses extension for Scoping Reviews.

* Where *sources of evidence* (see second footnote) are compiled from, such as bibliographic databases, social media platforms, and Web sites.

† A more inclusive/heterogeneous term used to account for the different types of evidence or data sources (e.g., quantitative and/or qualitative research, expert opinion, and policy documents) that may be eligible in a scoping review as opposed to only studies. This is not to be confused with *information sources* (see first footnote).

‡ The frameworks by Arksey and O’Malley (6) and Levac and colleagues (7) and the JBI guidance (4, 5) refer to the process of data extraction in a scoping review as data charting*.*

§ The process of systematically examining research evidence to assess its validity, results, and relevance before using it to inform a decision. This term is used for items 12 and 19 instead of "risk of bias" (which is more applicable to systematic reviews of interventions) to include and acknowledge the various sources of evidence that may be used in a scoping review (e.g., quantitative and/or qualitative research, expert opinion, and policy document).

*From:* Tricco AC, Lillie E, Zarin W, O'Brien KK, Colquhoun H, Levac D, et al. PRISMA Extension for Scoping Reviews (PRISMAScR): Checklist and Explanation. Ann Intern Med. 2018;169:467–473. [doi: 10.7326/M18-0850](http://annals.org/aim/fullarticle/2700389/prisma-extension-scoping-reviews-prisma-scr-checklist-explanation).
